# *Cryptococcus albidus* meningitis: A case report and literature review

**DOI:** 10.1097/MD.0000000000042125

**Published:** 2025-04-25

**Authors:** Genzhu Wang, Hang Liu, Xiaoying Wang, Zhongdong Li

**Affiliations:** a Beijing Electric Power Hospital of State Grid Co. of China, Capital Medical University Electric Teaching Hospital, Beijing, China.

**Keywords:** case report, central nervous system infection, cryptococcal meningitis, *Cryptococcus albidus*, liposomal-amphotericin B, review

## Abstract

**Rationale:**

Central nervous system (CNS) infections caused by *Cryptococcus albidus* are rarely reported, but are often associated with high mortality rates. Clinical data on the appropriate use of liposomal-amphotericin B (liposomal-AmB) with cryptococcal meningitis are limited. Here, we report for the first time the efficacious and safe use of the ideal bodyweight to calculate the dose of liposomal-AmB for a severely obese patient with *C albidus* meningitis. We also review the cases of CNS infection caused by *C albidus* and its related species.

**Patient concerns:**

A severely obese female patient was admitted to neurosurgical ward with lesions in the intracranial space and accompanying headache. Brain magnetic resonance imaging revealed prominent lesions in the right parietal and left frontal insular region. After glioma resection, *C albidus* was isolated from her cerebrospinal fluid samples.

**Diagnoses, interventions, and outcomes:**

She diagnosed histopathologically with a *Cryptococcus* species infection. The culture from her cerebrospinal fluid sample showed the growth of yeast-like colonies, which were identified as *C albidus* with mass spectrometry. She was treated with liposomal-AmB (50 mg day^-1^ intravenously) plus 5-fluorocytosine (10 g day^-1^ orally in 4 divided doses) for 6 weeks. The patient was asymptomatic at the time of discharge.

**Lessons:**

CNS infections of *C albidus* were uncommon. Using ideal bodyweight to calculate the dose of liposomal-AmB should be considered for severely obese patients with *C albidus* meningitis.

## 1. Introduction

Central nervous system (CNS) infections of *Cryptococcus* species are life-threatening diseases. *C albidus*, also called *Naganishia albida*, was first reported to infect humans in 1965,^[1]^ and several opportunistic infections associated with *C albidus* have since been reported.^[2–4]^ To date, the yeast has been isolated from the cerebrospinal fluid (CSF), lung abscesses, pleural fluids, and blood specimens of patients.^[5]^ Of those invasive infections, 30% of the patients had a CNS infections.^[6]^ Most notably, the mortality rate was 67% in patients with CNS infections caused by *C albidus*, which is more than double that of patients infected with other *Cryptococcus* species.^[6]^ Although fewer than 10 cases of *C albidus* infection have been reported,^[1,7]^ the fungus has received attention because its mortality rate is high. Here, we report a clinical case of cryptococcal meningitis caused by *C albidus*. Clinically isolated *C albidus* may include many other related species, for example, *C adeliensis* and *C diffluent*.^[8]^ No clear-cut epidemiological markers have been described to determine the likelihood of *C albidus*. Recommended guidelines for its treatment are also lacking. Therefore, it is extremely important to identify the risk factors, clinical presentation, diagnosis, and treatment for CNS infection due to *C albidus* and its related species.

## 2. Case presentation

**Figure 1.**
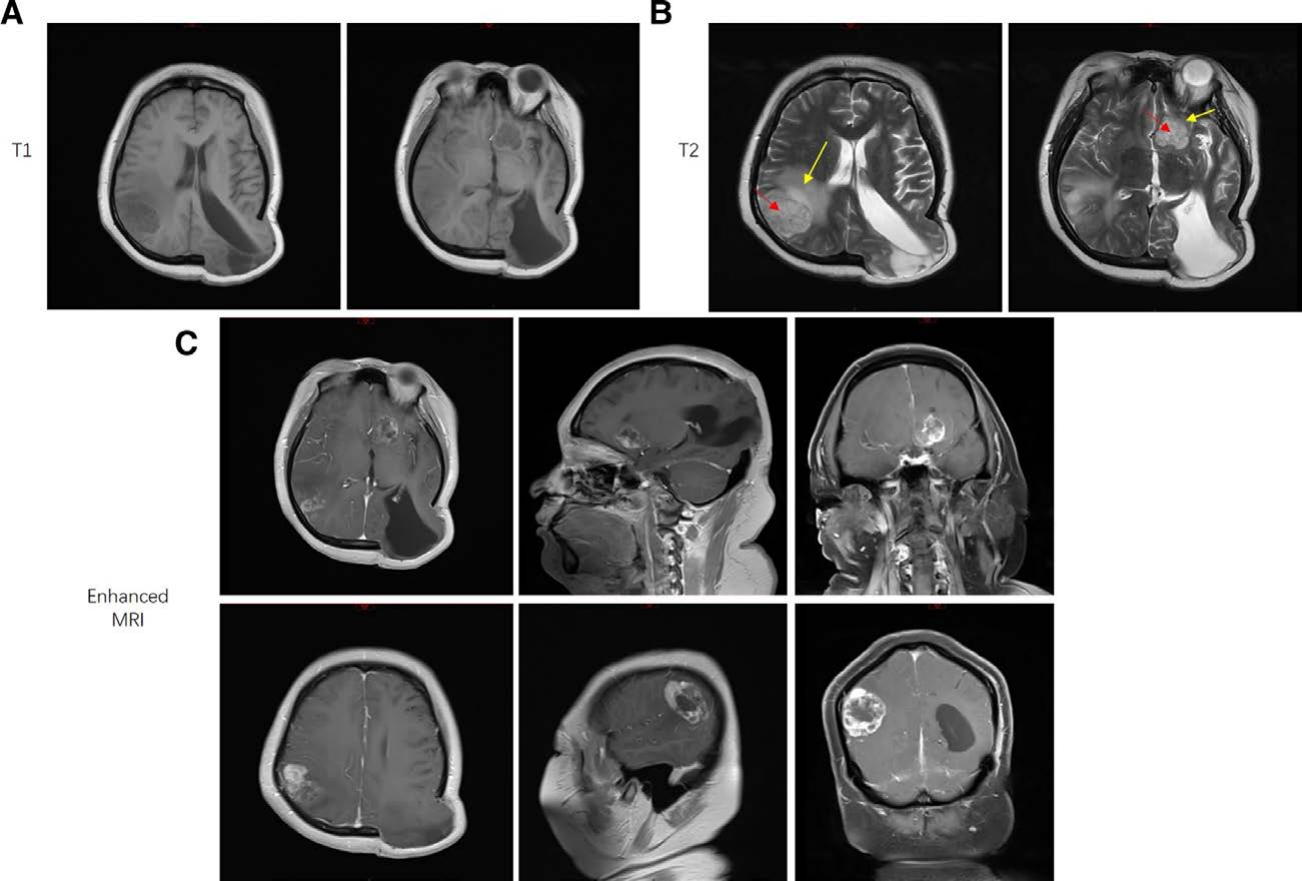
Brain magnetic resonance imaging (MRI) scan of the patient before surgery. A type of circular lesion was observed in the right parietal lobe with long signals on T1-weighted (A) and T2-weighted (B) images. The lesion was about 4 × 4 × 3 cm^3^ in size (red arrow) and accompanied by moderate edema of surrounding brain tissue (yellow arrow). Another type of circular lesion was deep in the left frontal lobe, with long signals on T1-weighted (A) and T2-weighted (B) images. The lesion was about 3 × 3 × 3 cm^3^ in size (red arrow) and accompanied by mild edema (yellow arrow). Enhanced MRI scanning showed that the lesions were unevenly and significantly enhanced (C).

A 25-year-old female patient (height was 158 cm, bodyweight was 100 kg) was admitted to Beijing Electric Power Hospital (Beijing, China) in March, 2022 with lesions in the intracranial space and accompanying headache. Six months before admission to the hospital, she had undergone successful left temporoparietal occipital craniotomy and decompressive craniectomy. Afterwards, she subsequently suffered intermittent headache, concurrently with nausea and disordered consciousness, and was treated with mannitol and levetiracetam before her admission. The patient denied any family or medical history, except invasive procedures of her CNS 6 months previously. She was a farmer, in frequent contact with cats and dogs. A physical examination revealed no unusual findings. Specifically, her body temperature was 36 °C, blood pressure 130/70 mm Hg, pulse rate 68 beats/min, and respiratory rate 17 breaths/min. Brain magnetic resonance imaging revealed prominent lesions in the right parietal accompanied by moderate edema and left frontal insular region with mild edema. The lesion volume in the right parietal region and the left frontal insular was about 4 × 4 × 3 cm^3^ and 3 × 3 × 3 cm^3^, respectively (Fig. 1).

On day 0 (admission), the patient’s associated laboratory data were normal. Specifically, her white blood cell count was 9.5 × 10^9^ L^-1^; neutrophils, lymphocytes, and monocytes accounted for 73.8%, 21.1%, and 4.9%, respectively. Her red blood cell count was 4.4 × 10^12^ L^-1^, hemoglobin level was 133.0 g L^-1^, and platelet count was 213.0 × 10^9^ L^-1^. Urinalysis, hepatic and renal function tests were normal. During hospitalization, no chemotherapy and/or other immunosuppressive drug was administered. On day 5 after admission, the patient underwent right parieto-occipital for glioma resection. However, her headache persisted.

In the postoperative period, the patient experienced seizures, treated with mannitol and valproic acid. On day 14, she was diagnosed histopathologically with a *Cryptococcus* species infection. On day 16, the culture from her CSF sample showed the growth of yeast-like colonies, which were identified as *C albidus* with VITEK mass spectrometry (BioMerieux, France). To date, no Clinical and Laboratory Standards Institute clinical breakpoints or epidemiological cutoff values have been established for *C albidus*. The susceptibility of the pathogen to 5 antifungal compounds was tested in accordance with the guidelines of Clinical and Laboratory Standards Institute document M27-A3.^[9]^ The minimal inhibitory concentrations of amphotericin B (AmB), fluconazole, voriconazole, itraconazole, and 5-fluorocytosine for *C albidus* were determined to be 0.5, 4, 1, 0.125, and 4 mg L^-1^, respectively.

**Figure 2.**
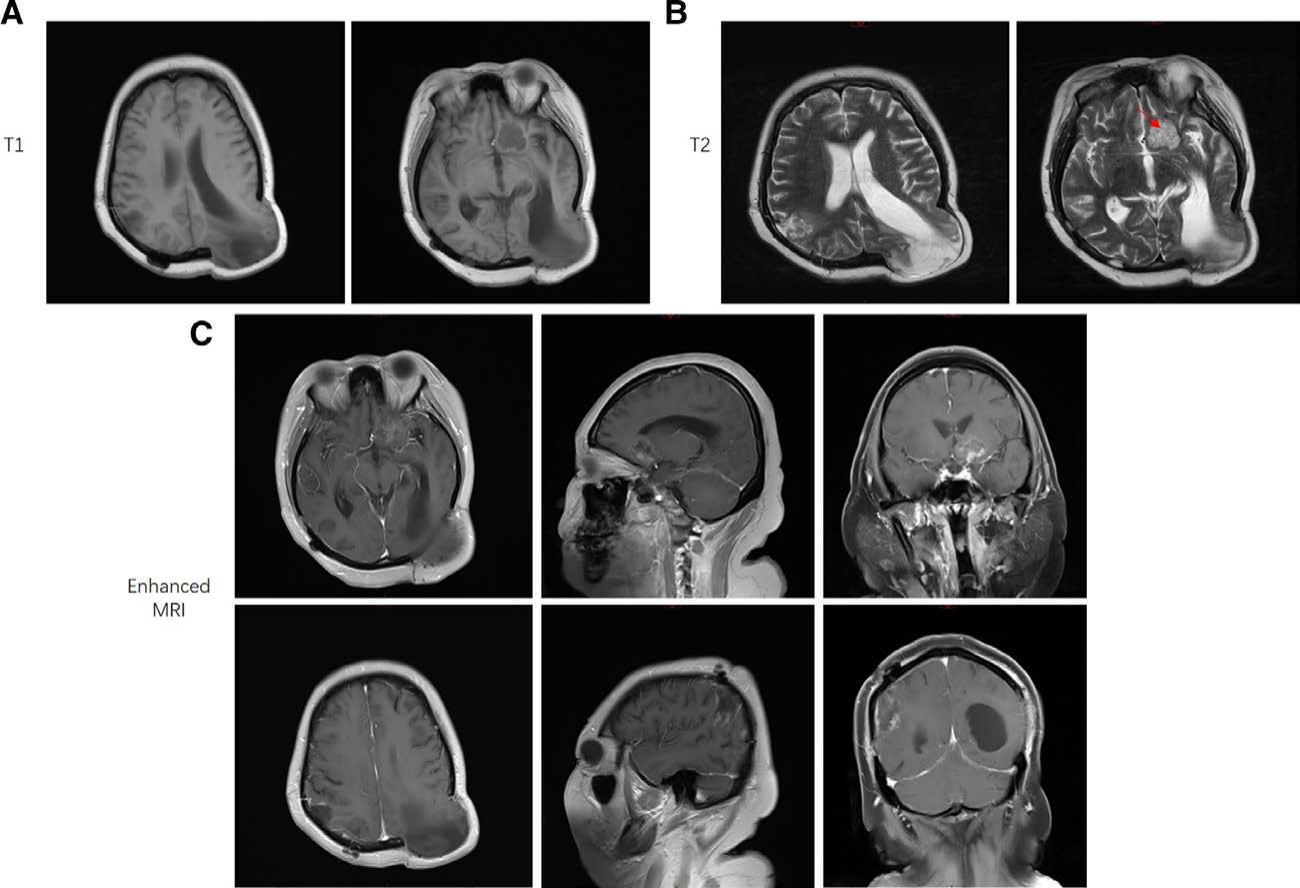
MRI scan of the patient after surgery. The lesion in the right parietal lobe was resected, whereas the lesion in the left frontal lobe (red arrow) was still observed with long signals on T1-weighted (A) and T2-weighted (B) images. Enhanced MRI scanning showed that the lesion was only mildly enhanced after treatment (C).

**Figure 3.**
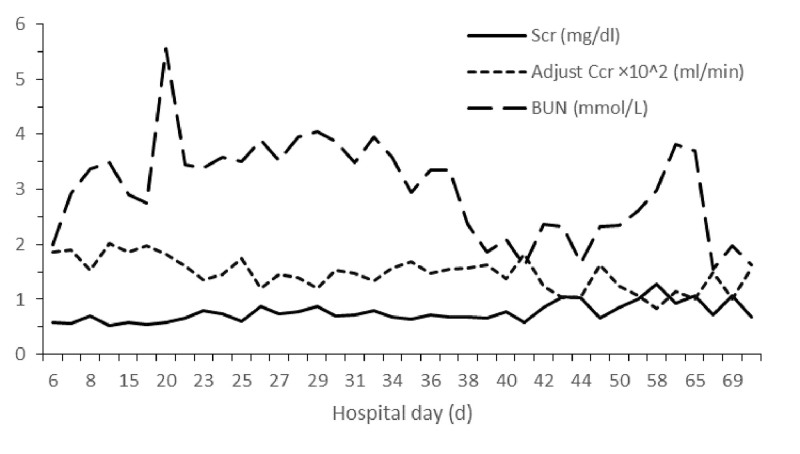
Renal function of the patient. Adjusted creatinine clearance rate (Ccr) was calculated with the following equation: Ccr (mL/min) = (146 - age) × (0.287 × weight [kg] + 9.74 × height^2^ [m]^2^)/ (60 × serum creatinine[mg/dL]). BUN = blood urea nitrogen, Scr = serum creatinine.

After the patient was diagnosed with a *Cryptococcus* species infection, she was administered with of liposomal-AmB (50 mg day^-1^ intravenously) qd plus 5-fluorocytosine (10 g day^-1^ orally in 4 divided doses) for 6 weeks. Her magnetic resonance imaging results showed that only the left frontal insular region still had a lesion, with otherwise normal parenchyma, after 3 weeks of treatment (Fig. 2). In addition, no yeast was isolated from 3 different CSF samples after 4 weeks of treatment (Table 1). The patient maintained normal renal function during the entire treatment period (Fig. 3). On day 48 after admission, the patient stopped complaining of headache, and suffered no further nausea or consciousness disorder. The patient was asymptomatic at the time of discharge, with normal glucose, protein, and lactate levels in the CSF.

**Table 1:** Analysis of cerebrospinal fluid.

Hospitalization days	Glucose	Chloride	Microalbumin	Color	Turbidity	Pandy test	Red blood cells	White blood cells	Neutrophil	Lymphocyte	Culture
	mmol/L	mmol/L	mg/L				/mm^3^	/mm^3^	%	%	
3/16	2.88	118.2	80	Colorless	Clear	Positive	0	106	22	78	
3/21	2.32	123	95	Colorless	Slightly turbid	Positive	1000	339	64	36	
3/22	2.09	114.4	133	Red	Turbid	Positive	56,000	1196	46	54	*C albidus*
3/30	2.22	129.1	59	Colorless	Slightly turbid	Slightly positive	0	104	25	75	
4/03	4.07	130.2	50	Colorless	Clear	Positive	0	149	1	99	Negative
4/19	3.99	127.6	46	Colorless	Clear	Negative	1000	136	2	98	Negative
4/21	4.05	132.1	28	Colorless	Clear	Negative	0	26	4	96	Negative

## 3. Discussion

*C albidus* in the genus *Cryptococcus*, is a rare species that causes cryptococcal infections, especially in the CNS infections. The antifungal treatment recommended for cryptococcal meningoencephalitis is liposomal-AmB plus flucytosine.^[10,11]^ Although liposomal-AmB has been used for several decades, little is known about its pharmacokinetics in obese patients.^[12,13]^ The prescribing information for cryptococcal disease recommends weight-based doses of 0.7 to 6 mg/kg/day AmB, depending upon the AmB product used. To date, the calculation of the appropriate dose for severely obese patients is controversial.^[13,14]^ Animal studies have demonstrated significantly increased AmB serum concentrations and increased rates of nephrotoxicity in obese rats and rabbits, with poor distribution of adipose tissue.^[15]^ Furthermore, women have a higher body fat rate than men, which is a risk factor for acute kidney injury caused by liposomal-AmB.^[16]^ Therefore, it may be reasonable to consider the ideal bodyweight rather than the actual bodyweight when calculating the dose of liposomal-AmB for severely obese female patients. In this case, we have demonstrated the efficacy and safety of using the ideal bodyweight, rather than the actual bodyweight, to calculate the dose of liposomal-AmB for a severely obese patient with *C albidus* meningitis.

A comprehensive review of the literature was performed in accordance with the “Preferred Reporting Items for Systematic Reviews and Meta-Analysis.”^[17–19]^ Studies of case report relevant to CNS infections attributed to *C albidus* and its related species in the PubMed, Medline, and Web of Science databases from their inception until July 2024. The following key words were used: fungus, human infection, CNS, meningitis, ventriculitis, encephalitis, non-neoformans, cryptococcus, naganishia, adeliensis, albida, albidus, chernovii, curvatus, diffluens, friedmannii, liquefaciens, magnus, terreus, uniguttulatus, and uzbekitanensis. The references in each manuscript were also reviewed to identify additional cases.

Our searches yielded 104 potentially relevant records. After nonhuman species (e.g., dog, cat, pigeon) were excluded,^[20,21]^ 86 studies were included. After non-CNS cases, non-research studies, non-*Cryptococcus, C neoformans* species complex, and *C gattii* species complex infections were excluded,^[10,22–28]^ 17 studies were reported. Of those 17 studies, 7 were cohort studies of *Cryptococcus*,^[11,29–34]^ and 1 study was of *C laurentii* meningitis combined with *C albidus* cryptococcaemia.^[35]^ Finally, 9 cases of CNS infections caused by *C albidus* or its related species have been reported. The risk factors, manifestations, diagnosis, and treatment for these pathogens are presented in table 2. Like other *Cryptococcus* species, *C albidus* and its related species were commonly isolated from the gastrointestinal tracts and droppings of birds.^[2,36]^ There is, as yet, no explanation of how humans contract these infections. Reasons may include the worldwide increase in immunocompromised patients, the widespread use of immunosuppressive agents, and the prolonged use of central venous catheters or indwelling devices.^[9]^ In our systematic review of the literature, we detected 2 cases of infection in human immunodeficiency virus (HIV) infected adults and 4 in apparently immunodeficient individuals.^[3,7,9,38]^ Leukemia and cancer are important risk factors in HIV-negative patients.^[36,37,39]^ The administration of corticosteroids agents, exposure to bird excrement, contact with animals, and surgery are also risk factors for these infections.^[2,3,36]^

**Table 2:** Summary of data from central nervous system infection caused by *C albidus* and its related species in humans.

Author, year	Country	Age/gender	Potential risk factors	Manifestation	Duration of symptoms	*Cryptococcus* species	Taxonomical identification methods	Treatment	Outcomes
da Cunha Lusins (1973)^[2]^	New York, USA	45/M	Exposure to pigeon excrement	Meningitis, headache, nausea and vomiting	3 days	*C albidus*	Culture	AmB (1500 mg)	Cured
Melo et al (1980)^[3]^	Kentucky	29/M	Mentally retarded, receiving corticosteroids agents, surgery	Meningitis, headache	2 weeks	*C albidus*	Culture	AmB	Died
McCurdy et al (2001)^[36]^	Nashville, USA	65/F	Ovarian cancer, invasive neurosurgical procedure	Ventriculitis, headache and mental status changes.	1 day	*C uniguttulatus*	Culture and ID 20C	AmB (0.6 mg/kg) + 5-FU	Cured
Rimek et al (2004)^[37]^	Germany	40/F	Acute myeloid leukemia, peripheral blood stem cell transplantation and central venous catheter	Meningitis, fever, headache and paraparesis	4 days	*C adeliensis*	Culture, ID 32C and ITS D1/D2 Sequencing	Liposomal-AmB (5 mg/kg) + AmB (0.25 mg intrathecally biw) + 5-FU (120 mg/kg)	Died
Pan et al (2011)^[9]^	China	37/M	Low CD4 lymphocyte count	Meningitis, fever, cough, vomiting and headache	15 days	*C uniguttulatus*	Culture and ITS D1/D2 Sequencing	AmB (0.7 mg/kg) + 5-FU (100 mg/kg qid)	Cured
Liu et al (2013)^[7]^	China	28/M	AIDS	Encephalitis, diplopia, vomiting, tinnitus, neck stiffness, strabism and vertigo.	2 weeks	*C albidus*	Culture	Fluconazole (NA)	Died
Conde-Pereira et al (2015)^[38]^	Guatemala	31/F	AIDS	Meningitis, headache, vomiting, fever, asthenia and adynamia	1 month	*C liquefaciens*	Culture, ID 32C, MALDI-TOF MS and ITS D1/D2 Sequencing	AmB deoxycholate (0.7 mg/kg)	Died
Animalu et al (2015)^[39]^	Memphis, USA	72/F	Non-small-cell lung carcinoma	Meningitis, headache, nausea and emesis	unknown	*C uniguttulatus*	Culture and ID 20C	Liposomal-AmB (5 mg/kg) + fluconazole (400 mg bid)→ voriconazole	Cured
Present case	China	25/F	Glioma, surgery	Meningitis, headache, vomiting and nausea	6 months	*C albidus*	Culture and VITEK MS	Liposomal Amp B (50 mg qd) + 5-FU (100 mg/kg qid)	Cured

→ = change to, AIDS = acquired immunodeficiency syndrome, AmB = amphotericin B, F = female, 5-FU = fluconazole, ITS = internal transcribed space, M = male, MALDI-TOF MS = matrix-assisted laser desorption ionization-time of flight mass spectrometry, MS = mass spectrometry.

Patients can present with headache, fever, altered mental state, nausea, and vomiting. Headache is the commonest symptom, and only 1 patient reported no headache.^[7]^ Although fever is possible, it only presented in 3 cases (31%). These results are similar to a cohort study that showed fever present in 28% of cases.^[29]^ Patients also presented with increased intracranial pressure increasing and altered mental states, and these neurological symptoms are similar to other cryptococcal infections.^[28]^ The cryptococcal capsule lodges in the arachnoid villi and subsequently prevents CSF reabsorption may explain the elevated intracranial pressure.^[40]^

Culture and API® strip assimilation test are laboratory commonly used methods to identify isolated *Cryptococcus* species.^[7,38,39]^ However, it may be difficult to identify species when only 1 or 2 methods are used. Without the sequence of the D1/D2 domains of the large-subunit of ribosomal RNA (rRNA), some rare *Cryptococcus* species can be misidentified as *C albidus*.^[8,27,41]^ For example, a yeast isolate was initially identified as *C albidus* with an API® strip assimilation test. However, after the internal transcribed spacer and the D1/D2 domains of the large-subunit rRNA were sequenced, the isolate was finally confirmed as *C liquefaciens*.^[38]^ Mass spectrometry is a new methodology for identifying bacterial species, and some studies have shown that the accuracy of mass spectrometry for their identification is over 95% at the species level.^[42–44]^ Hence, the identification of *C albidus* based on VITEK mass spectrometry is reliable.

Because these fungi are infrequent causes of disease, there are no evidence-based guidelines or standard recommendations for their treatment. The susceptibility of *C albidus* and its related species to antifungal agents is shown in Table 3. The cumulative experience reported in the published literature suggests that AmB is the only agent to which *C albidus* and its related species are consistently sensitive.^[45,46]^ Therefore, AmB is a safe and effective drug for use as initial induction therapy against these infections in the CNS. However, consideration should be given henceforth to submitting these clinical isolates for susceptibility testing and proper identification to guide subsequent therapeutic decisions. To date, no randomized controlled trials have been conducted to optimize the choice of AmB or the duration of its therapy. We recommend: (1) using liposomal-AmB instead of AmB deoxycholate because liposomal-AmB is safer and more rapidly sterilizes the CSF than AmB deoxycholate^[11,12,32]^; (2) at least 1 g of AmB during treatment and prolonged treatment are recommended.^[47]^ (3) The combination of AmB and 5-fluorocytosine, is recommended because it has been shown to reduce the toxicity of this drug and reduce the relapse rate of *C albidus* infections.^[30,31]^

**Table 3:** Antifungal susceptibility of *Cryptococcus* species.

Antifungal agents	Minimal inhibitory concentrations (μg/mL)					
	McCurdy et al (2001)	Rimek et al (2004)	Pan et al (2011)	Conde-Pereira et al (2015)	Animalu et al (2015)	Present case
	*C uniguttulatus* ^[36]^	*C adeliensis* ^[37]^	*C uniguttulatum* ^[9]^	*C liquefaciens* ^[38]^	*C uniguttulatus* ^[39]^	*C albidus*
Amphotericin B	0.25	0.125	0.125	1	≤0.12	0.5
5-Flucytosine	>64	>64	>64	>64	64	4
Fluconazole	>64	32	64	>256	128	4
Voriconazole	NG	0.25	0.5	>8	0.25	1
Itraconazole	1	0.25	1	>16	0.5	0.125
Posaconazole	NG	NG	0.5	>8	1	NG

## 4. Conclusion

Using the ideal bodyweight to calculate the appropriate dose of liposomal-AmB for obese patients with *C albidus* meningitis is both efficacious and safe. Based on the systematic review of CNS infections of *C albidus* and its related species, we also concluded that leukemia and cancer are important risk factors for these infections and that headache is the most frequent symptom. The identification of fungal pathogens requires the analysis the rRNA sequences because many species share phenotypic similarities. AmB-based treatment should be the first choice, and subsequent treatment should be guided by susceptibility testing.

## Acknowledgments

We thank the patient for participating in this study. We thank International Science Editing (http://www.internationalscienceediting.com) for editing this manuscript. We thank Professor Yiyou Gu (The Lundquist Institute at Harbor-UCLA Medical Center, Torrance, California, USA) for review services and Professor Xiaorui Wang (Beijing Electric Power Hospital) for critical reading of the MRI scan results.

## Author contributions

**Conceptualization:** Zhongdong Li.

**Data curation:** Hang Liu, Xiaoying Wang.

**Methodology:** Hang Liu.

**Writing – original draft:** Genzhu Wang.

**Writing – review & editing:** Xiaoying Wang, Zhongdong Li.
